# Data on reducing carbon footprint in microgrids using distributed battery energy storage

**DOI:** 10.1016/j.dib.2019.104679

**Published:** 2019-10-19

**Authors:** Javad Khazaei, Colton Schlauderaff

**Affiliations:** a Pennsylvania State University, United States; b Penn State Harrisburg, United States

**Keywords:** Battery energy storage systems, Microgrids, CO_2_ emissions, Power systems, Smart grids

## Abstract

This data presented in this article was collected using simulations on a microgrid system to analyze reduction of carbon footprints using distributed battery storage devices. Analysis was performed over a 24-h period of operation of the microgrid system to reduce the CO_2_ emissions from 0% to 100% using battery storage devices. The data can be used in designing efficient microgrid systems, understanding the potential of battery energy storage devices in future electricity generation, and sizing the microgrid systems depending of the CO_2_ reduction goals in power systems.

**Table:** Specifications Table

Subject	Electrical and Electronic Engineering Renewable Energy
Specific subject area	Application of Battery Energy Storage in Microgrids
Type of data	Table Graph Figure
How data were acquired	Time-domain Simulations using MATLAB Software
Data format	The data is in the form of “.slx” and “.m”, which are specifically for MATLAB simulations and MATLAB scripts, respectively.
Parameters for data collection	Data collection was done under normal operating condition of a microgrid system with diesel generator, solar photovoltaic (PV), battery energy storage devices, and various loads in a simulated microgrid model in MATLAB. The simulation was run for 24 hours of operation by scaling 1 hour to 1 second in the simulation and time step of 1e-5 second.
Description of data collection	The data were collected by running the simulated microgrid system in 5 different scenarios from no battery energy storage devices in the microgrid to 400-kilowatt (kW) battery storage systems, and various solar PV capacities (from 200 kW to 400 kW). The average diesel generator's fuel consumption was used for each scenario to calculate the amount of CO_2_ reduction in the overall microgrid system.
Data source location	Pennsylvania State University, Middletown, PA, U.S., Zip code: 17057.
Data accessibility	The data is available in Mendeley Data public repository. The link to the data is provided in the following. https://data.mendeley.com/datasets/3rmy9fzx32/1

**Table:** 

**Value of the Data** • The data helps scientific community to understand how to reduce CO_2_ emissions in microgrids. • Any scientist, engineer, or student who is interested in saving the climate, would benefit from the data presented in this article. Power plants are major cause of CO_2_ emissions in electricity generation units. By promoting the application of renewable energy sources to reduce the electricity generated in power plants (by burning fossil fuels), the climate change affect can be reversed. The presented data can help scientists, engineers, or students to size the renewable energy source or battery energy storage to achieve desired CO_2_ reduction levels. • The data can be used to develop hardware setups and actual microgrids for specific percentages of CO_2_ reduction in microgrids.

## 1 Data

**Fig. 1:**
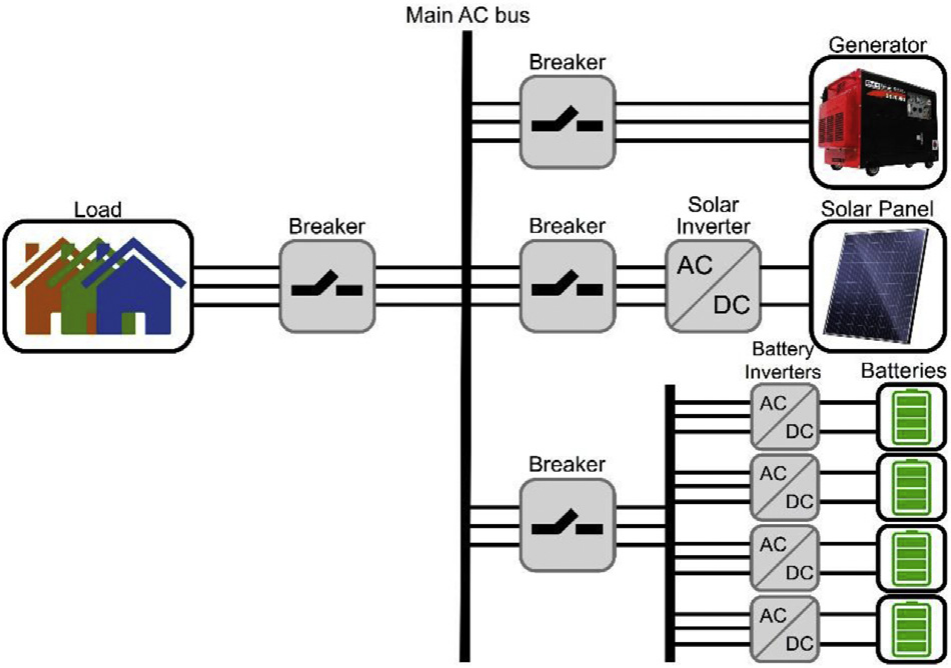
Structure of the simulated microgrid system.

Microgrids have become the most reliable sources of energy generation for future power systems [1]. The main structure of the test system is illustrated in Fig. 1. The microgrid system was simulated in MATLAB software, which included a 400 kW diesel generator, a variable 200–400 kW solar PV connected to the main AC bus using a DC/AC converter and a breaker, and 4 individual battery energy storage systems connected to the main AC bus through 4 standalone DC/AC bi-directional inverters. The fuel consumption or 24-h period is computed based on the percentage of the load from the generator's data sheet. For example, for 400kW load support by the generator, referring to Ref. [2], the generator consumes 27.8 gallons of fuel per hour for 100% load. Then, for the 24-h period, the fuel consumption in gallons per hour is multiplied by 24.

The system load was composed of fixed resistive load of 400 kW.

### 1.1 Case 1: No battery storage

**Fig. 2:**
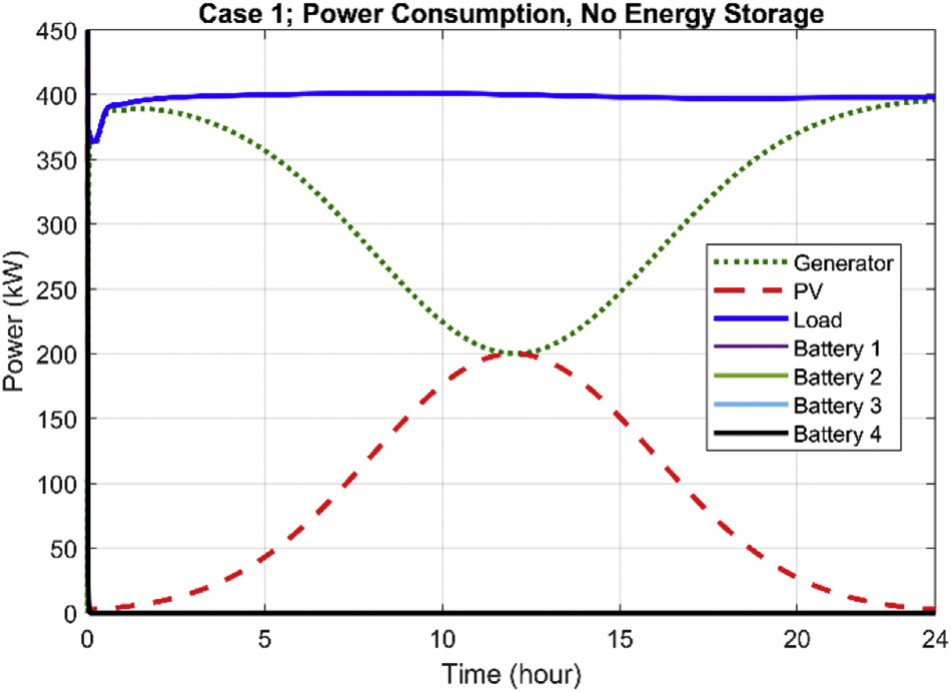
Daily generation profile of the microgrid system without battery storage.

**Fig. 3:**
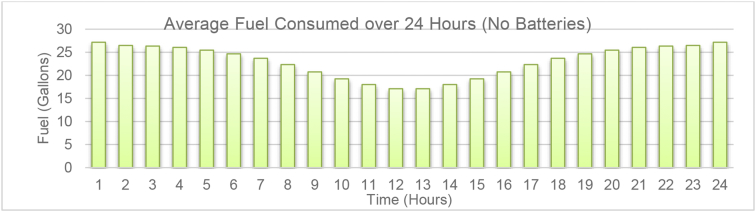
Average fuel consumption of the diesel generator during the 24-h period of operation.

Fig. 2 illustrates the 24-h generation profile of the microgrid system with no battery energy storage devices. As it can be observed, the batteries do not produce any active power and the diesel generator cooperates with solar PV to provide a fixed 400 kW power to the load over a 24-h period. As it can be confirmed in Fig. 2, the solar generation provides its maximum output power (200 kW) in a sunny day (assumption of the simulation) between 11 a.m. and 1 p.m. During this period, the generator's power is at its minimum (200 kW), which reduces the CO_2_ emissions significantly. The average fuel consumption rate (per gallon) for the 24-h operation of the system without any battery energy storage is illustrated in Fig. 3. As it is observed, the fuel consumption significantly reduces when the solar generation is at its maximum (11 a.m.–1 p.m.).

### 1.2 Case 2: with battery storage

In this case scenario, the system is enhanced with 4 individual battery energy storage systems, as was previously shown in Fig. 1. Each battery storage is rated at 50 kW and can support constant 50 kW over 24-h operation of the system.

**Fig. 4:**
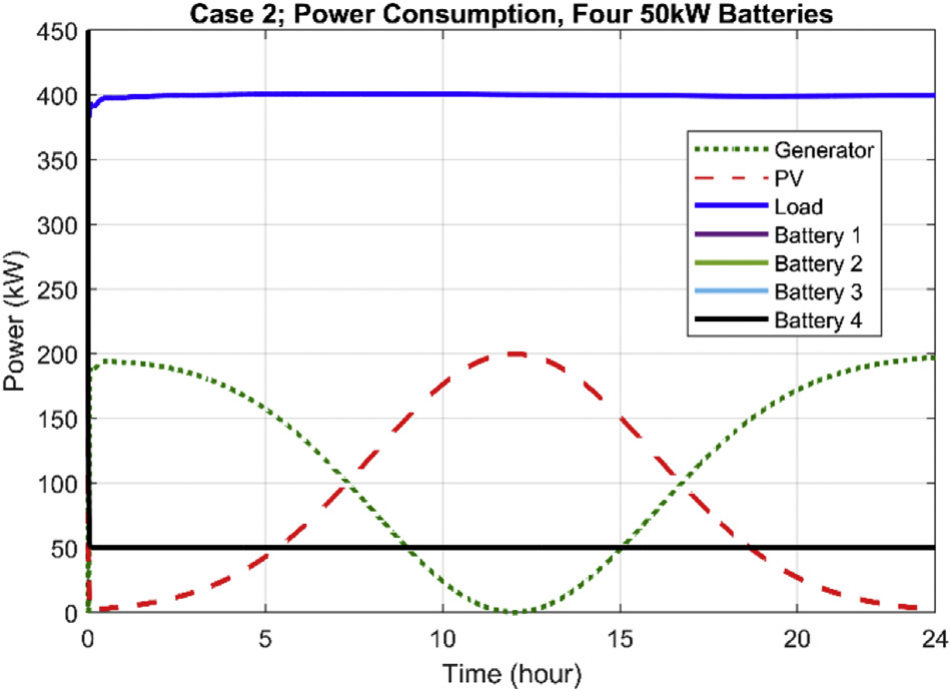
Daily generation profile of the system with batteries.

Simulation data for this case are illustrated in Fig. 4. As it can be seen, the batteries (all four) support 50 kW of load power over the 24-h period of operation. Therefore, 200 kW of the load power is supported by the batteries. The remaining 200 kW of the load demand is shared between the generator and the solar PV. As it can be seen, during the 11–1 p.m. operating period of the system, the PV generation is at its maximum, and the overall load demand (400 kW) is supported by PV and batteries, therefore, the generator's power drops to zero. Consequently, the amount of released CO_2_ reduces.

**Fig. 5:**
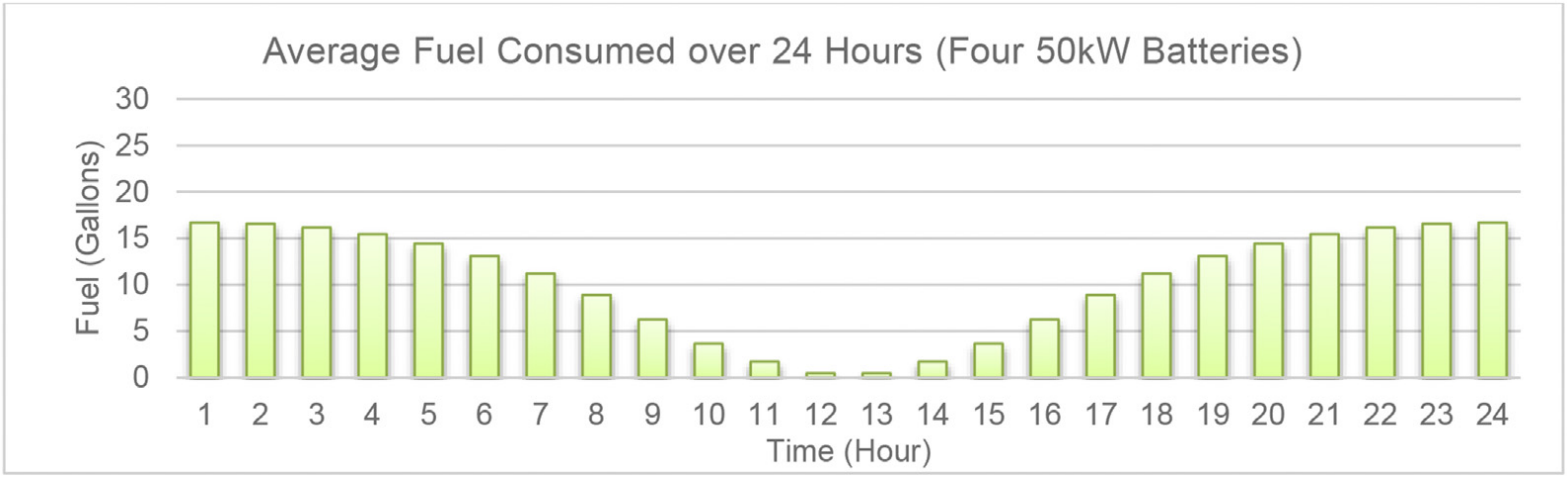
Average fuel consumption for 24-h period of the operation for case 2.

Fig. 5 illustrates the average fuel consumption per gallon for the diesel generator in the system for the 24-h period of the operation when all the batteries are in operation. Compared to the first scenario (see Fig. 3), the average fuel consumption has significantly reduced due to the concurrent operation of distributed energy storage devise in the system. It is also observed that the fuel consumption or CO_2_ emissions have significantly reduced during 11–1 p.m. period, as was anticipated in Fig. 4.

### 1.3 Case 3: nonlinear relationship between fuel consumption and output power of generators

**Fig. 6:**
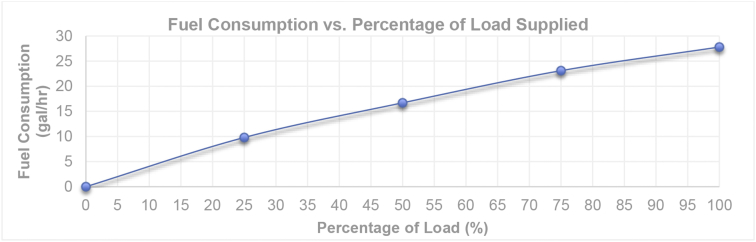
Average fuel consumption per percentage of load supplied by the diesel generator.

Fig. 6 depicts the relationship between the average fuel consumption of diesel generators at gallon per hour rate versus percentage of the demand supplied by the generator. As it can be seen, a 400 kW generator consumes about 27 gallons of fuel per hour to support a 400 kW load. However, this relationship is not linear, for example, the same generator consumes 10 gallons per hour to support 25% of the demand as illustrated by Fig. 6. Therefore, one should consider the nonlinear relationship of fuel consumption of generators versus the output power when designing a microgrid system.

### 1.4 Case 4: CO_2_ emissions reduction

In this case, the amount of fuel needed for various CO_2_ emission reduction levels for 24-h period of operation are considered in five scenarios. In the first case, the CO_2_ reduction is set to 0%, where all the power is provided by the generator. For the second case, the fuel consumption will be reduced by 11.18%, in the third case, it is reduced to 23.69%, in the fourth case, it is reduced to 38.54% and finally, 55% fuel consumption reduction is achieved in case 5. Data is illustrated in Table 1; as it can be seen, the total gallons of fuel needed for 24-h operation of the system as well as total CO_2_ emissions in kilogram (kg) are shown in Table 1. The CO_2_ emission rate is considered as 11.36 kg CO_2_ per gallon of fuel [3].

**Table 1:** Total gallons needed for CO_2_ reduction levels in 5 scenarios.

	Case 1	Case 2	Case 3	Case 4	Case 5
Total gal fuel	554.5076	492.482	423.1252	340.7912	249.1256
Total kg CO_2_	6,297	5,593	4,805	3,870	2,829
Reduction	0%	11.18%	23.69%	38.54%	55.07%

### 1.5 Case 5: energy storage capacity needed for CO_2_ reduction levels

In this case, different CO2 reduction levels are considered, and the amount of battery energy storage needed to achieve those reduction level with their respected capacity is elaborated. The data for this case is shown in Table 2. For example, as it can be seen, the 20% CO_2_ reduction requires about 85 kW average storage rated at about 2-GW hour (GWh). Similarly, for a 50% CO_2_ reduction in the test system, 185 kW storage at the capacity of 4.4 GWh is needed.

**Table 2:** Total energy storage needed over 24 hours.

CO_2_ Reduction	Average (kW)	Capacity (MWh)
0%	0	0
10%	44.5874	1070.098
11.18%	50	1200
20%	85.2199	2045.278
23.69%	100	2400
30%	122.2934	2935.042
38.54%	150	3600
40%	155.8079	3739.39
50%	185.7634	4458.322
55.07%	200	4800
60%	212.1599	5091.838
70%	234.9974	5639.938
80%	254.2759	6102.622
90%	269.9954	6479.89
100%	282.1559	6771.742

### 1.6 Case 6: cost analysis

The last scenario details a cost analysis of various options to reduce CO_2_ emissions through various energy storage capacities and solar rated powers. The Data is shown in Table 3. The cost for the entire solar PV system per kW was considered a $3,050 before 30% tax credit reduction [3], the diesel generator const for a 400kW diesel generator was considered as $60,470 [4], the diesel generator fuel was considered $3.17 per gallon [5], and Lithium-ion energy storage battery cost was considered $209 per kWh [6]. The data can easily be updated for different costs on a yearly basis. It is observed that the most affordable case is when there is no battery energy storage in the system, however, as was discussed in previous scenarios, the CO_2_ emissions are at their maximum when there is not battery energy storage in the system. However, once the battery is implemented (case 4 for example) the system cost would be double, however, the CO_2_ emission reductions are significant (see previous scenarios).

**Table 3:** Cost analysis of various options in the microgrid system to reduce CO_2_ emissions.

	Case 1	Case 2	Case 3	Case 4
	Solar: 200kW Generator: 400kW 0 Batteries	Solar: 200kW Generator: 400kW 1 Battery: 50kW, 1,25 MWh each	Solar: 200kW Generator: 400kW 2 Batteries: 100kW, 1.25 MWh each	Solar: 200kW Generator: 400kW 3 Batteries: 150kW, 1.25 MWh each
Solar system	$427,000.00	$427,000.00	$427,000.00	$427,000.00
Diesel generator	$60,470.00	$60,470.00	$60,470.00	$60,470.00
Diesel fuel	$1,757.79	$1,561.17	$1,341.31	$1,080.31
Batteries	$0.00	$260,832.00	$521,664.00	$782,496.00
Total	$489,227.79	$749,863.17	$1,010,475.31	$1,271,046.31
	Case 5	Case 6	Case 7	Case 8
	Solar: 200kW Generator: 400kW 4 Batteries: 200kW, 1.25 MWh each	Solar: 200kW 4 Batteries: 1,99 MWh each	Solar: 300kW 4 Batteries: 1,73 MWh each	Solar: 400kW 4 Batteries: 1,46 MWh each
Solar system	$427,000.00	$427,000.00	$640,500.00	$854,000.00
Diesel generator	$60,470.00	$0.00	$0.00	$0.00
Diesel fuel	$789.73	$0.00	$0.00	$0.00
Batteries	$1,043,328.00	$1,663,640.00	$1,446,280.00	$1,220,560.00
Total	$1,531,587.73	$2,090,640.00	$2,086,780.00	$2,074,560.00

## 2 Experimental design, materials, and methods

**Fig. 7:**
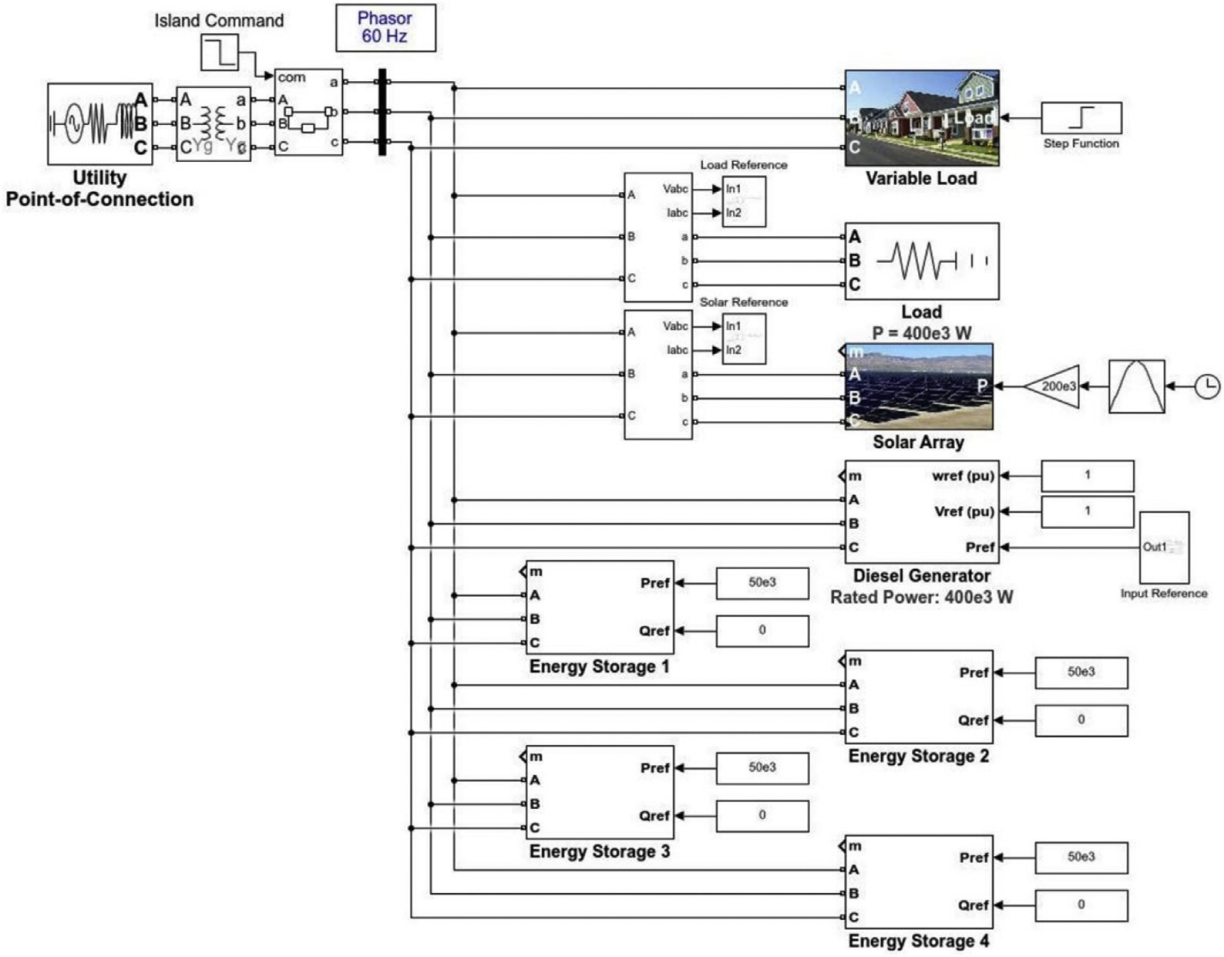
Simulated model in MATLAB software.

The simulated model was designed in MATLAB Simpower System toolbox, where a detailed tutorial for developing microgrids in MATLAB is included in Ref. [7]. The simulated model is illustrated in Fig. 7. The system is composed of a fixed 400 kW load, a solar array, which receives input irradiance and generates the output AC power using a DC/AC converter. The model was available in Microgrid library [7], and the input was generated using a probability distribution function (PDF) block in MATLAB library. The diesel generator was also available in the library and the inputs (voltage and frequency) was set to 1 per unit. The energy storage modules were also available in microgrid library [7] and their inputs were modified based on available power (mismatch between the load power and total generation (synchronous generator and solar PV)). The entire system was connected to the main grid (ideal voltage source) at the beginning and a breaker was used to isolate the system from the grid after 0.01 seconds.
